# Analysis of transcription factor- and ncRNA-mediated potential pathogenic gene modules in Alzheimer’s disease

**DOI:** 10.18632/aging.102169

**Published:** 2019-08-16

**Authors:** Cuihua Zou, Jie Wang, Xiaohua Huang, Chongdong Jian, Donghua Zou, Xuebin Li

**Affiliations:** 1 Department of Neurology, Youjiang Medical University for Nationalities, Baise, Guangxi 533000, People’s Republic of China; 2 Department of Nephrology, Youjiang Medical University for Nationalities, Baise, Guangxi 533000, People’s Republic of China; 3 Department of Neurology, The Fifth Affiliated Hospital of Guangxi Medical University, Nanning, Guangxi 533022, People’s Republic of China

**Keywords:** Alzheimer’s disease, differential expression analysis, modularization, protein-protein interaction network

## Abstract

Alzheimer’s disease (AD) is a progressive neurodegenerative disease that ranks as the fourth most common cause of death in developed countries. In our study, genes differentially expressed between AD and healthy individuals were identified and used to construct protein-protein interaction (PPI) networks. The AD-related PPI network was used to identify functional modules, and enrichment analysis showed that they were significantly involved in “Alzheimer’s disease”, “apoptosis”, and related pathways. We predicted non-coding RNAs and transcription factors that may regulate the functional modules. The expression of hub genes and transcription factors was validated in an independent data set. The results in this study provide several candidates for further research on mechanisms of AD pathogenesis.

## INTRODUCTION

As the global population ages, prevalence of Alzheimer’s disease (AD) and associated mortality increases, which places tremendous pressure on the families of patients and burdens the healthcare system. The symptoms include inattention, defects in working memory, and impairment of executive function and information processing. The most common neuropsychiatric symptom in patients is apathy [1]. Peripheral symptoms include depression, cognitive impairment, urinary incontinence, and inflammation. This disease is characterized by the presence of amyloid-beta plaques and neurofibrillary tangles [2].

Several genes have been associated with higher risk of AD, including CR1, CD33 and TREM2 [3, 4]. Some non-coding RNAs (ncRNAs) and transcription factors (TFs) also play important regulatory roles in the disease, including microRNA-200a [5], microRNA-200a-3p [6], MALAT1 [7], and microRNA-186 [8]. AD is a complex disease involving multiple genes and signaling cascades. Given the complexity of the disease, understanding its pathogenesis will require studies of multiple gene modules on a global level.

As a step in this direction, the present study constructed a protein-protein interaction (PPI) network based on genes differentially expressed between AD and healthy individuals. This network was then used to mine functional modules of target genes as well as the ncRNAs and TFs that regulate them.

## RESULTS

**Figure 1:**
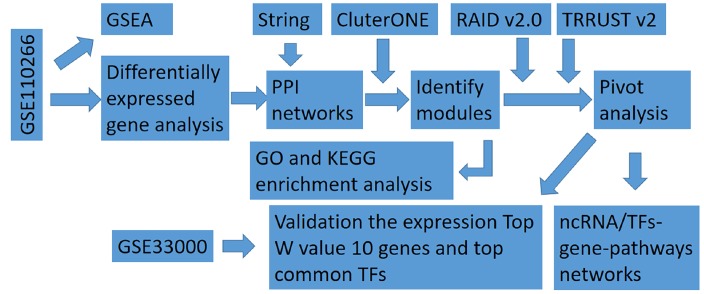
**Flowchart in this study.** GSEA, gene set enrichment analysis; ncRNA, non-coding RNA; PPI, protein-protein interaction; TF, transcription factor.

The steps of this study are shown in Figure 1, and clinical information for the dataset GSE110226 for identification of genes differentially expressed in AD is shown in Supplementary Table 1.

### Gene set enrichment analysis

**Figure 2:**
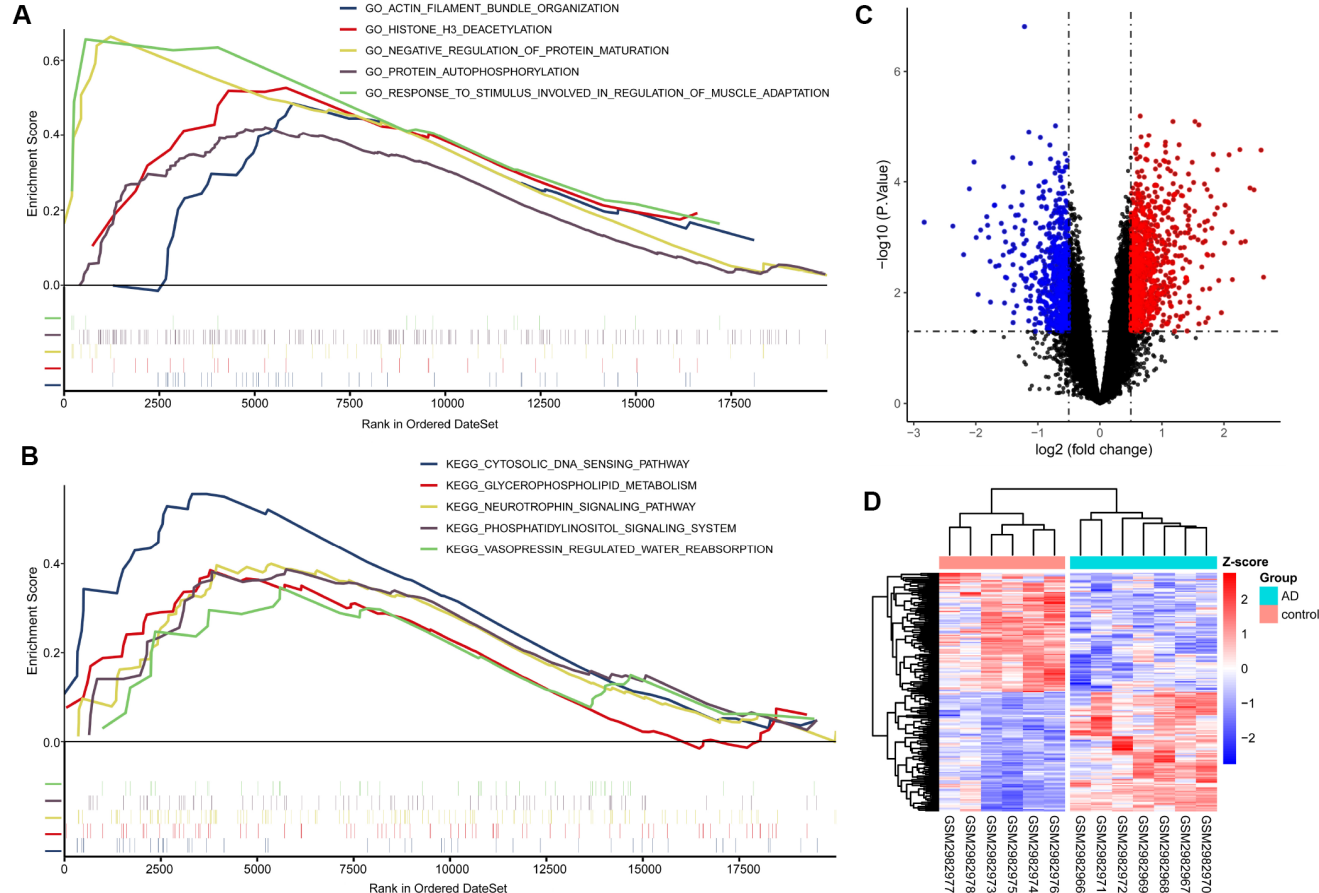
**GSEA, difference analysis and cluster analysis.** (**A**) Five of the most significantly enriched BP gene ontology (GO) terms. (**B**) Five KEGG pathways with the most significant enrichment. The mini vertical line indicates genes. (**C**) Volcanic maps of differentially expressed genes. Red indicates genes upregulated in AD; blue, genes downregulated in AD. (**D**) Cluster analysis heatmap showing how these expression patterns of these DEGs can distinguish AD from normal control tissues.

This analysis suggested that AD samples were significantly enriched in protein regulation-related biological processes, such as “negative regulation of protein maturation” and “protein autophosphorylation.” KEGG pathway analysis indicated that AD samples were significantly enriched in neurotrophic pathways, such as phosphatidylinositol and neurotrophin signaling (Figure 2A–2B).

### Differentially expressed genes and cluster analysis

A total of 4239 differentially expressed genes (DEGs) were identified in the GSE110226 dataset, of which 2542 were up-regulated and 1697 were down-regulated in AD (Figure 2C). Cluster analysis was performed with the most 100 upregulated DEGs and 100 most downregulated DEGs. Cluster analysis showed that the expression pattern of these 200 DEGs could accurately distinguish AD from control samples (Figure 2D).

### PPI network and its modular analysis

**Figure 3:**
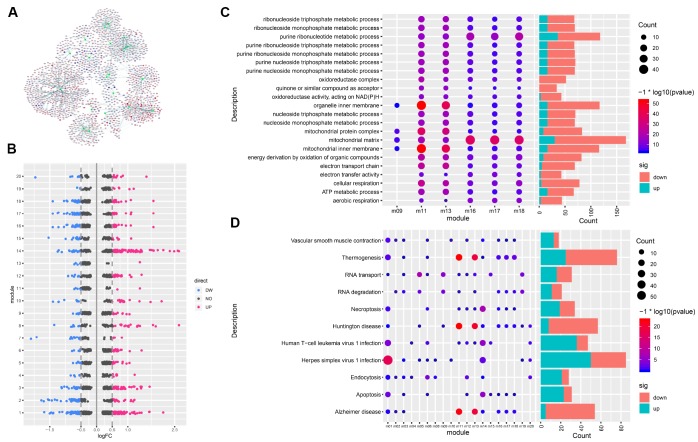
**Gene modules and functional enrichment.** (**A**) Gene modules and related genes. The green node indicates the module; red, genes upregulated in AD; blue, genes downregulated in AD. (**B**) Differentially expressed genes in each module. (**C**) GO Enrichment Analysis. Enrichment increases significantly going from blue to red. The larger the circle, the more significant the proportion of module genes present among GO functional entry genes. (**D**) Enrichment analysis of KEGG pathway of the module gene. From blue to red, the enrichment increases significantly. The larger the circle, the more significant the proportion of module genes present among KEGG pathway entry genes.

A PPI network of DEGs was constructed with 3861 gene nodes and 268363 edges using the STRING v10 database. The weight (W) value of nodes in the network was defined as | logFC |* - log_10_ (P value) * Degree. The larger the W value, the more critical the node is in the PPI network. The gene nodes with the highest W values in the network were SLC11A1, SERPINE1, EFCAB3, PIM1, IL6, BCL6, RND3, ZBTB16, LRG1, and RASL10B (Supplementary Table 2). These were considered as hub genes. Using the ClusterONE plug-in cohesion-guided algorithm, we excavated 20 functional modules containing 1730 related genes (Figure 3A, 3B).

In order to explore the role of functional modules in the pathogenesis of AD, we performed GO function and KEGG pathway enrichment analysis for each module. Results of GO function enrichment (Supplementary Table 3) indicated GO terms for 2114 biological processes, 296 cell components, and 393 molecular functions, while pathway enrichment analysis identified 1203 KEGG pathways (Supplementary Table 4). We found that six modules were significantly enriched in the GO terms of mitochondrial inner membranes and mitochondrial matrix. Figure 3C shows the GO terms in which more than four modules were significantly enriched, and Figure 3D shows the KEGG pathways involving more than eight modules. Any or several of these 20 functional modules may work together to form a functional pathway contributing to AD.

### Module-related ncRNAs and TFs

**Figure 4:**
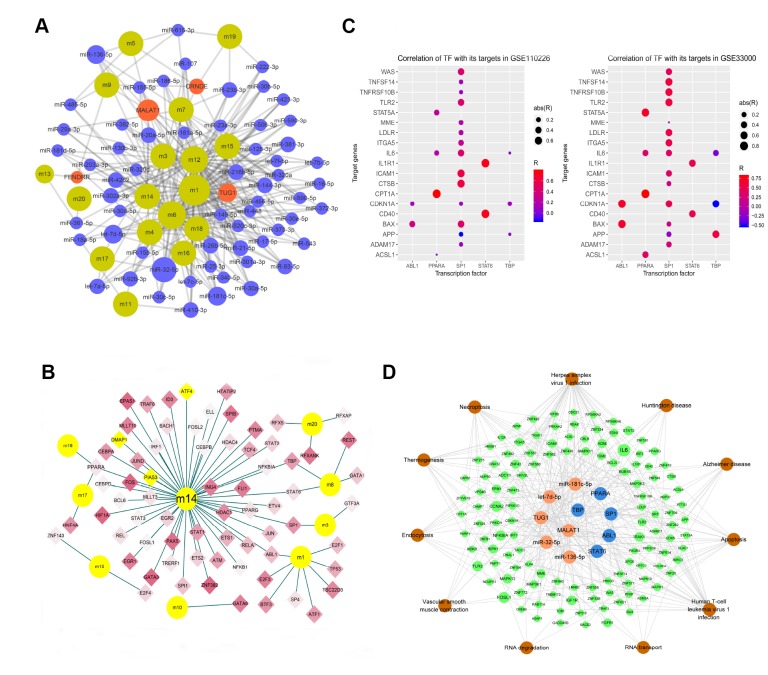
**Modular network regulation map of gene-related ncRNA/TFs.** (**A**) Map of gene module regulation by ncRNAs. Brown indicates modules; red, long non-coding RNA; and blue, microRNA. The size of the node reflects the node's degree. (**B**) Map of modular genes and the TFs regulating them. Yellow dots indicate modules; diamonds, transcription factors; red, genes upregulated in AD; and blue, genes downregulated in AD. Yellow diamond nodes indicate expression that is not significantly different between AD and control samples. (**C**) Correlation of TFs with their targets. Abbreviations: abs, absolute value; R, Pearson correlation coefficient. (**D**) Integrated regulatory network of ncRNA/TF-target genes-pathways. Orange indicates non-coding RNA; blue, TF; green, module gene; and brown, pathway.

The hypergeometric test predicted 706 ncRNAs participating in 1198 pairs of ncRNAs and target functional modules. MicroRNA-32-5p may regulate eight functional modules, MALAT1 may regulate seven, while let-7d-5p, TUG1, microRNA-136-5p, and microRNA-181c-5p may regulate six (Figure 4A).

The hypergeometric test predicted 70 TFs involved in 77 pairs of TFs and target functional modules. These TFs were differentially expressed in AD to varying degrees (Figure 4B). PPARA was predicted to regulate three functional modules, while ABL1, SP1, STAT6, and TBP were predicted to regulate two modules.

These results suggest that six ncRNAs and five TFs may be strongly associated with AD pathogenesis. We performed correlation analysis of the five TFs with their target genes in order to reduce noise and false positives (Figure 4C), and the resulting significant correlations were used to build a network. Combining this network with KEGG enrichment analysis allowed us to construct an AD-related ncRNA/TF-target genes-pathways integrated regulatory network (Figure 4D).

### Validation of differential expression and ROC analysis

**Figure 5:**
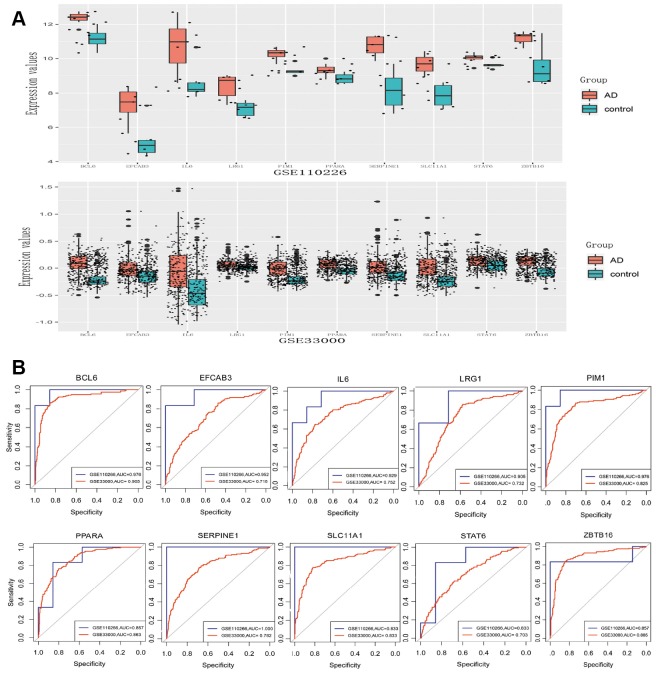
**Differential expression validation and ROC analysis of 8 differentially expressed genes and two transcription factors.** (**A**) Expression in GSE110226. (**B**) Expression in GSE33000. (**C**) ROC analysis in GSE110226.

The expression of genes with the Top 10 W values and the five TFs mentioned above were validated using the GSE33000 data set. Eight of the 10 genes (BCL6, EFCAB3, IL6, LRG1, PIM1, SERPINE1, SLC11A1, ZBTB16) and two TFs (PPARA and STAT6) were significantly up-regulated in AD (p < 0.05), consistent with the analysis of GSE110226 (Figure 5A). Analysis of receiver operating characteristic (ROC) curves suggested that these molecules may be potential biomarkers for AD diagnosis (Figure 5B). This may be especially true for BCL6 in the GSE110226 dataset (area under the ROC curve, 0.976) and GSE33000 dataset (area under the ROC curve, 0.905).

## DISCUSSION

AD is a neurodegenerative disease characterized by progressive dementia, neuroinflammation, intracellular neurofibrillary tangles and accumulation of extracellular plaques. There appear to be four main causes: the hypothesis of abnormal folding and aggregation of amyloid-beta/tau protein, activation of the innate immune system, mitochondrial dysfunction and oxidative stress [9]. In this study, we collected the gene expression profiles and normal control brain tissues of AD in GSE110226 from GEO. We identified genes differentially expressed between AD [10] and healthy controls based on the GSE110226 dataset, and we constructed a PPI network. The PPI networks revealed 20 functional modules related to AD.

Enrichment analysis suggests that the functional modules are involved in multiple GO terms and pathways, which likely reflects the complexity of the disorder. We observed that there were six functional modules enriched in the mitochondrial inner membrane and mitochondrial matrix. Mitochondria are inhibited by Ca^2+^ signaling. Excessive production of Ca^2+^ and reactive oxygen species induce the opening of the mitochondrial transition pore mPTP, causing the loss of mitochondrial function and cell death, ultimately leading to AD. Abnormalities of mitochondria have been associated with aging and age-related neurodegenerative diseases such as cancer, diabetes, AD, Parkinson's disease, amyotrophic lateral sclerosis and Friedrich ataxia [11].

Potential ncRNA and TF regulators involved in AD-related functional modules were predicted using the hypergeometric test. The predicted AD-related TFs were confirmed to be abnormally expressed in AD. MicroRNA-32-5p was predicted to regulate eight functional modules; MALT1, seven modules; and let-7d-5p, microRNA-136-5p, microRNA-181c-5p and TUG1, six modules. MicroRNA-32-5p inhibits TR4 expression by binding to the 3' untranslated region of its transcript. The resulting deficiency of TR4 alters transcription of genes involved in HGF/Met signaling [12]. The long ncRNA MALAT1 participates in basic cellular processes, including epigenetics, transcription and post-transcriptional regulation of gene expression. Altering levels of MALAT1 affects brain development as well as neuronal function and maintenance in neurodegenerative diseases [13]. MALAT1 inhibits expression of BAX, caspase-3 and Bcl-2 as well as the p-PI3K/p-mTOR/p-GSK3beta signaling pathway, thereby promoting apoptosis of A_beta_-induced human neuroblasts [14]. MicroRNA-181c may bind to the 3' untranslated region in the transcript encoding collapsing response mediator protein 2 (crmp2), which allows it to regulate axon orientation, MAPK signaling, dorsoventral axis formation, and long-term depression in neuronal signaling. Dysregulation of crmp2 abundance can lead to AD-related dysfunction [15]. MicroRNA-181 regulates c-Fos and SIRT-1 proteins and affects synaptic plasticity and memory processing in AD mice [16]. Let-7d-5p, for its part, binds to the RNA polymerase II promoter, increases p53 signal transduction and positively regulates microRNA transcription [17], thereby causing AD dysfunction. All these results identify several candidates that may regulate multiple functional modules to contribute to AD and therefore may be interesting therapeutic targets. We describe the first integrated regulatory networks involving ncRNA/TFs and target genes in functional modules that may contribute to AD.

The hypergeometric test identified 70 differentially expressed TFs that may regulate AD functional modules. PPARA may regulate three modules, while ABL1, SP1, STAT6, and TBP may regulate two modules each. Consistent with our findings, the Epistasis project identified four significant interactions between single nucleotide polymorphisms in PPARA and SNP in IL-1A, IL-1B, and IL-10 that were associated with higher AD risk [18]. SP1 can regulate gene FE65, which act as a ligand of Alzheimer’s disease amyloid precursor protein, and SP1 can promote the expression of SNAP-25, which is involved in the pathogenesis of neuropsychiatric disorders, including schizophrenia, attention deficit hyperactivity disorder and AD [19–21]. STAT6 activates amyloid-beta 42 production in the brain of adult zebrafish, increasing the proliferation and neurogenesis of nerve stem/progenitor cells (NSPCs) involved in AD. In addition, TATA-binding protein can accumulate in the brain of AD patients, leading to formation of neurofibrillary tangles, which can cause onset of AD [22].

We validated the top W values of 10 genes in the PPI network and five TFs based on another data set. Eight genes and two transcription factors were significantly upregulated in the GSE33000 dataset. Analysis of the area under ROC curves suggests that these molecules may be biomarkers for AD diagnosis, especially BCL6. BCL6 appears to be absent from neurofibrillary tangles associated with AD plaques [23], so future studies should examine its role in AD.

Our results should be interpreted with caution in light of some limitations. Firstly, though the hub genes and TFs were validated in the large dataset GSE33000, the validation dataset GSE110226 was relatively small. Secondly, our studies were limited to in silico predictions, so our findings should be verified and extended in laboratory experiments. Indeed, our predictions were based on analyses of post-mortem samples, so they should be validated in vivo, especially the differential expression of hub genes. Follow-up studies should also clarify whether the predicted AD-associated ncRNAs and TFs activate or inhibit their corresponding functional modules, which our *in silico* studies could not determine.

## MATERIALS AND METHODS

### Data resources

We collected the set of gene expression profiles from AD from the Gene Expression Omnibus database (GSE110226) [24, 25]. This dataset includes post-mortem brain samples from 7 patients with AD and 6 healthy individuals. This dataset was obtained using a Rosetta/Merck Human RSTA Custom Affymetrix 2.0 microarray [HuRSTA-2a520709]. We constructed PPIs of DEGs based on human PPI data in the STRING V10 database [26]. Then, we screened pairs of interacting ncRNA-mRNAs in the RAID v2.0 database [27] and identified 43,1937 interaction pairs involving 5,431 ncRNAs that scored at least 0.5. Data on 2492 human transcription factors (TFs) and 9396 TF-gene interaction pairs were downloaded from the TRRUST V2 database [28].

### GSEA analysis

The GSE110266 gene expression profile was downloaded and normalized using the “quantile” method by normalizing between array functions in the *limma* package [29–31]. We screened biological process GO terms and KEGG pathways that may be related to AD using GSEA (GSEA2-2.2.4, Java version) [32, 33]. The datasets c5.bp.v6.2.symbols.gmt and c2.cp.kegg.v6.2. symbols.gmt in the MsigDB V6.2 database [34] were used as reference gene sets, and GSEA was performed according to default parameters. We set NOM P < 0.05 as the threshold for significant enrichment.

### Identification of DEGs and cluster analysis

DEGs between AD and control samples were identified from pre-GSEA normalized expression profiles using the lmFit and eBayes functions in the *limma* package [29–31]. Differences associated with an unadjusted P < 0.05 were considered significant. We also screened the data using a threshold of a false discovery rate-adjusted p < 0.05, but we found that numerous genes with biological functions potentially relevant to AD were missed (data not shown). Two-way hierarchical clustering was performed on DEG expression profiles based on Euclidean distance, and the results were shown as a heatmap.

### PPI network construction and recognition module

We constructed a PPI network of DEGs based on the STRING V10 database and visualized it using Cytoscape software [35]. Then we used the Cytoscape plug-in ClusterONE [36] to predict protein complexes based on a cohesion algorithm and nearest neighbor selection. The higher the cohesion score in the ClusterONE algorithm, the more likely it is that the interacting proteins form a complex. We visualized DEGs in functional modules using Cytoscape.

### GO function and KEGG pathway enrichment analysis

To help identify the potential functions of the genes in AD-associated modules, we used the clusterProfiler package [37] in R to perform enrichment analysis of the 20 modules according to gene ontology (GO) functions (p-value cutoff = 0.01, qvalueCutoff = 0.01) and KEGG pathway (p-value cutoff = 0.05, qvalue Cutoff = 0.2). ClusterProfiler is an R package of Bioconductor, which can perform statistical analysis and visualization of functional clustering of gene sets or gene clusters.

### Identification of ncRNAs and TFs in regulatory modules

Interactions between ncRNAs and their target genes were downloaded from the RAID v2.0 database, and interactions between TFs and their target genes were downloaded from the TRRUST v2 database. Interactions between a regulator and its target functional module were examined using the hypergeometric test in the R program. Interactions between a regulator and a functional module that showed quantity >2 and P<0.01 were considered significant. We also analyzed correlation between the TF and its targets in order to reduce noise and false positives, although most interactions between TF and target in the TRRUST database have been validated.

### Validation of differential expression and modular common TFs and ROC analysis

Independent gene expression profiles (GSE33000) containing AD and healthy brain tissue were obtained from the Gene Expression Omnibus and used to validate the 10 DEGs with the highest W values, as well as TFs predicted to regulate more than two functional modules. The data set GSE33000 contained 310 AD cases and 157 healthy brain tissues. In these two data sets, ROC analysis was carried out to evaluate the ability of these genes to differentiate AD from healthy controls. The pROC package [38] was used for ROC analysis.

## CONCLUSIONS

We identified AD-related functional gene modules and ncRNAs and TFs that regulate them, providing candidate molecules for further study of AD.

## Supplementary Material

Supplementary Table 1

Supplementary Table 2

Supplementary Table 3

Supplementary Table 4
