# Supplementary material for: Analysis of transcription factor- and ncRNA-mediated potential pathogenic gene modules in Alzheimer’s disease
Source: Aging (Albany NY). 2019 Aug 16;11(16):6109–19. doi: 10.18632/aging.102169 (PMC6738443; doi:10.18632/aging.102169)
Supplement: Supplementary Table 1 [file aging-11-102169-s001.pdf]

Supplementary Table 1. Clinical information for GSE110226.

Clinical information for GSE110226							
**Samples**	**Age**	**Sex**	**Apoe genotype**	**ba_rin**	**ba8s18s**	**disease state**	**Braak staging**
GSM2982966	74	female	E4/E4	4.1	0.648	AD	III-IV
GSM2982967	84	female	E3/E4	6.5	0.826	AD	severe V-VI
GSM2982968	84	male	E3/E4	6.6	0.814	AD	severe V-VI
GSM2982969	84	female	E3/E4	6.9	1.007	AD	severe + Lewy body disease
GSM2982970	89	male	E2/E3	6.8	0.947	AD	severe V-VI
GSM2982971	73	male	E3/E3	6.1	0.855	AD	severe V-VI
GSM2982972	70	male	E3/E4	7	0.953	AD	severe V-VI
GSM2982973	62	male	E3/E4	6.9	0.841	Control	
GSM2982974	55	female	E3/E3	7.6	1.197	Control	
GSM2982975	37	male	E3/E3	6.9	1.036	Control	
GSM2982976	64	male	E3/E3	7.2	1.12	Control	
GSM2982977	69	female	E3/E3	6.9	0.997	Control	
GSM2982978	70	male	E3/E4	5.8	0.636	Control	
